# Garden Cress Seed Oil Abrogates Testicular Oxidative Injury and NF-kB-Mediated Inflammation in Diabetic Mice

**DOI:** 10.3390/ijms242015478

**Published:** 2023-10-23

**Authors:** Rasha Abu-Khudir, Gehan M. Badr, Heba Ibrahim Abd El-Moaty, Rabab S. Hamad, Najla K. Al Abdulsalam, Aml Sayed Ali Abdelrahem, Saleha Alqarni, Mayyadah Abdullah Alkuwayti, Sherine Abdel Salam, Hanaa F. Abd El-Kareem

**Affiliations:** 1 Department of Chemistry, College of Science, King Faisal University, P.O. Box 380, Al-Ahsa 31982, Saudi Arabia; 2 Department of Chemistry, Biochemistry Division, Faculty of Science, Tanta University, Tanta 31527, Egypt; 3 Department of Zoology, Faculty of Science, Ain Shams University, Cairo 11566, Egypt; gbadr@sci.asu.edu.eg (G.M.B.); hanaafathy@sci.asu.edu.eg (H.F.A.E.-K.); 4 Department of Biological Sciences, College of Science, King Faisal University, P.O. Box 380, Al-Ahsa 31982, Saudi Arabia; hitorkey@kfu.edu.sa (H.I.A.E.-M.); rhamad@kfu.edu.sa (R.S.H.); najlaks@kfu.edu.sa (N.K.A.A.); malkwaiti@kfu.edu.sa (M.A.A.); 5 Medicinal and Aromatic Plants Department, Desert Research Center El-Mataria, Cairo 11753, Egypt; 6 Central Laboratory, Theodor Bilharz Research Institute, Giza 12411, Egypt; 7 Department of Nursing, College of Applied Medical Science, King Faisal University, P.O. Box 380, Al-Ahsa 31982, Saudi Arabia; aabdelrahem@kfu.edu.sa; 8 Department of Clinical Nutrition, College of Applied Medical Science King Faisal University, P.O. Box 380, Al-Ahsa 31982, Saudi Arabia; saalqarni@kfu.edu.sa; 9 Department of Zoology, Faculty of Science, Alexandria University, Alexandria 21511, Egypt; sherine.abdel.salam@alexu.edu.eg

**Keywords:** *Lepidium sativum* L. seed oil, diabetes mellitus, testis, oxidative stress, inflammation, histopathology

## Abstract

Diabetes mellitus is a metabolic disorder associated with various complications encompassing male reproductive dysfunction. The present study aimed to investigate the therapeutic potential of biologically active *Lepidium sativum* seed oil (LSO) against the testicular dysfunction associated with streptozotocin (STZ)-induced diabetes. Male adults (n = 24) were divided into four groups: control, LSO-administered, diabetic (D), and LSO-treated diabetic (D+LSO) groups. LSO was extracted from *L. sativum* seeds, and its chemical composition was determined using GC-MS. Serum testosterone levels, testicular enzymatic antioxidants (catalase (CAT) and superoxide dismutase (SOD)), an oxidative stress (OS) biomarker, malondialdehyde (MDA), pro-inflammatory markers (NF-kB, IL-1, IL-6, and TNF-α), and the expression level of NF-kB were assessed. In addition, histopathological changes were evaluated in testicular tissues. The results obtained showed that the chemical composition of LSO indicated its enrichment mainly with γ-tocopherol (62.1%), followed by 2-methylhexacosane (8.12%), butylated hydroxytoluene (8.04%), 10-Methylnonadecane (4.81%), and δ-tocopherol (3.91%). Moreover, LSO administration in the D+LSO mice significantly increased testosterone levels and ameliorated the observed testicular oxidative damage, inflammatory response, and reduced NF-kB expression compared to the diabetic mice. Biochemical and molecular analyses confirmed the histological results. In conclusion, LSO may prevent the progression of diabetes-induced impairment in the testes through inhibition of the OS- and NF-kB-mediated inflammatory response.

## 1. Introduction

Diabetes mellitus (DM) is an intricate metabolic disorder that results in persistent high blood glucose levels or hyperglycemia [1]. Being linked to an increased risk of micro- and macrovascular diabetic complications, prolonged hyperglycemia is generally associated with lower quality of life and responsible for the morbidity and mortality assigned to diabetes [2,3]. Multiple body organs are affected by micro- and/or macrovascular diabetic complications, resulting in their damage, dysfunction, and eventual failure [4,5]. Reproductive dysfunction is one of the adverse effects of diabetes in both humans and animal models [6,7,8,9]. Recently, it was reported that type 1 and type 2 diabetes (T1DM and T2DM, respectively) adversely affected the sperm quality and histopathology of reproductive organs in T1DM and T2DM mice with a great extent of similarity. However, the severity of reduced sperm concentration and precocious acrosome exocytosis were significant in T1DM compared to T2DM animals [10]. A substantial number of male diabetic subjects of reproductive age experience infertility, where the prevalence of infertility in diabetic men ranges from 35% to 51% [11,12,13]. In DM patients, various mechanisms are involved in the etiology of male infertility, including pre-, testicular, and post-testicular ones. The involvement of such mechanisms may differ depending on the type of diabetes (T1DM or T2DM), disease duration, and metabolic compensation of glycemic impairment [6]. Pre-testicular mechanisms involve central as well as peripheral hypogonadism, leading to decreased serum levels of gonadotropins, including follicle-stimulating hormone (FSH) and luteinizing hormone (LH), and testosterone (T) [14,15]. Indeed, high incidence of hypogonadism has been previously reported among type 2 diabetic men [16,17,18,19]. Moreover, a large amount of pre-clinical and clinical evidence supports substantial association between DM-related metabolic derangements and reduced serum testosterone levels [20,21]. At the testicular level, changes resulting from DM encompass increased OS consequent to overproduction of ROS and eventual lipid peroxidation (LPO) in seminal fluid, DNA fragmented sperms, altered mitochondrial bioenergetics, and the formation of advanced glycated end-products (AGEs) [22,23,24,25]. Moreover, plausible post-testicular mechanisms associated with DM include male accessory gland infection/inflammation (MAGI) and erectile and/or ejaculatory dysfunction. These pathogenic mechanisms result in altered sperm parameters, damaged sperms, and/or premature or delayed ejaculation, anejaculation, or retro-ejaculation [26,27,28].

Glucose metabolism in Sertoli cells (SCs) is crucial for the normal development of germ cells [29,30]. In addition, it is involved in maintaining fundamental cell activity as well as the motility and fertilizing ability of mature sperms [31]. However, chronic hyperglycemia triggers the activation of many pathological pathways associated with the development of diabetic complications, including elevated production of advanced glycated end-products (AGEs), the polyol pathway, the protein kinase C (PKC) pathway, the hexosamine pathway, and mitochondrial damage [32,33]. Upregulation of these pathways results in the overproduction of free radicals, including reactive oxygen species (ROS) and the decreased efficiency of antioxidant defense systems, which eventually lead to oxidative stress (OS) and increased cell death [34,35,36]. Indeed, increased OS is one of the major sources of the hyperglycemia-induced triggers of diabetic complications [37,38,39]. In this regard, it has been reported that many of the diabetes-induced molecular changes, that greatly influence sperm quality and function, are attributed to the increase in OS [40,41,42]. In fact, the male reproductive system is extremely vulnerable to OS mostly due to the restricted antioxidant enzyme systems in spermatozoa as well as the enrichment of sperm cell membranes with polyunsaturated fatty acids (PUFAs), rendering sperms extremely sensitive to ROS assault which ultimately leads to lipid peroxidation [43]. Consequent production of lipid peroxides and the disruption of membrane integrity extremely affect the viability and function of sperm cells, resulting in impaired motility and reduced fertilization capacity [44]. In addition to lipid peroxidation, sperm motility as well as fertilization ability are adversely affected by the ROS-induced oxidative damage of the proteins and nuclear DNA of sperm cells [45,46]. Moreover, hyperglycemia-induced overproduction of ROS generation has the potential to exacerbate mitochondrial dysfunction, eventually resulting in mitochondrial OS which ultimately leads to the apoptosis of spermatozoa and reduced viability of sperms [47,48]. In addition to disruption of antioxidant and mitochondrial mechanisms, hyperglycemia-induced ROS overproduction triggers the production of downstream inflammatory factors. OS is known to modulate NF-kB signaling pathway, thus initiating the activation of downstream inflammatory signaling pathways, resulting in the release of inflammatory factors such as IL-1, IL-6, and TNF-α [49,50]. Besides OS, inflammation is one of the common influencing causes that trigger damage to the male reproductive system as a result of diabetic complications [51,52]. Many studies have stated that hyperglycemia-induced pro-inflammatory factors could influence testicular cells or tissues [23,53]. Moreover, they can stimulate ROS generation, including nitric oxide (NO), which prolongs inflammatory reactions and can affect spermatogenesis, sperm maturation, and diminish semen quality [54,55,56,57].

Previous research has shown that conventional treatment of diabetes, whether using insulin or oral hypoglycemic medications, can have serious side effects and is ineffective in preventing the onset of numerous complications related to diabetes [58]. Therefore, there is an urgent need to implement safe compounds with plausible protective effects against oxidative and inflammatory changes induced by diabetes. In this regard, several studies have demonstrated the benefits of various medicinal plants and their derived bioactive phytochemicals in the management of diabetes-induced male infertility [59,60,61,62]. *Lepidium sativum* L., also known as Garden cress/Hab El Rashad, is a comparatively fast-growing annual herb that belongs to the family Brassicaceae [63]. It is native to Southeast Asia and Northeast Africa, and it is cultivated all over the world nowadays [64]. *L. sativum* possesses numerous pharmacological effects, namely antioxidant and anti-inflammatory activities; hence, it is commonly used in traditional medicine worldwide [65,66]. Secondary metabolites such as flavonoids, tannins, glycosides, polyphenols, lectin, and the mucilage were revealed by a phytochemical analysis of *L. sativum* extract [67,68,69]. The chemical composition of the plant’s seeds encompasses, among others, essential oils, fatty oils, fatty acids, carbohydrates, proteins, vitamins, flavonoids, alkaloids, isothiocyanate, and glycosides [68,70]. Moreover, natural antioxidants, such as tochopherols, are enriched in the seed extract [64]. In the field of folk medicine, the leaves and seed oil of *L. sativum* are known for their role in the treatment of various inflammatory disorders, including diabetes mellitus, asthma, and hepatitis [71,72,73,74,75]. Regarding diabetes-induced adverse effects on the male reproductive system, it was shown that *L. sativum* seed extract, as an antioxidant, played a beneficial protective role against the STZ-induced histopathological damage of the prostate gland and epididymis by preventing OS [76,77]. Moreover, improvement of semen parameters, sperm function, and sexual behavior have been observed in diabetic mice due to the cooperative ameliorative effects of *L. sativum* and co-enzyme Q10 (CoQ10). Such protective effects are partially mediated, at least, via their regulatory actions on the HPG axis through the triggering of the overexpression of the gonadotropin-releasing hormone (GnRH) gene and elevation in the production or secretion of LH, FSH, and testosterone [78]. Nevertheless, the preventive effect of *L. sativum* seed oil (LSO) on the basal cellular and molecular parameters of the male reproductive system has not been demonstrated at present. Hence, we hypothesize that *L. sativum* may be a plausible therapeutic alternative to conventional therapy for male infertility. In fact, there are now no specific treatments for diabetic testis lesions. The current study aimed to investigate the chemical compounds of *L. sativum* seed oil (LSO) and its potential role in mitigating the progression of diabetes-induced testicular impairment in a STZ-diabetes-induced mouse model.

## 2. Results

### 2.1. Identification of the Bioactive Compounds in LSO by GC-MS Analysis

**Figure 1:**
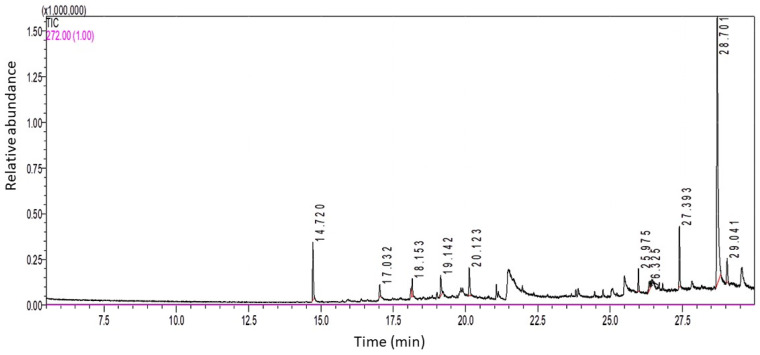
GC-MS spectral analysis of LSO.

Ten components representing 100% of the total fixed oil were identified in LSO. The GC-MS chromatogram in Figure 1 depicts peaks based on retention time (RT) and area %.

The data revealed high percentage of two forms of vitamin E (66.01%), including γ tocopherol (γT; 62.1%) and δ-tocopherol (δT; 3.91%) (Table 1). In addition, alkanes (24.22%), antioxidant phenol derivatives (8.04%), and alcohol (1.73%) were also detected.

### 2.2. Serum Testosterone Level in Diabetic Mice Treated with/without LSO

**Figure 2:**
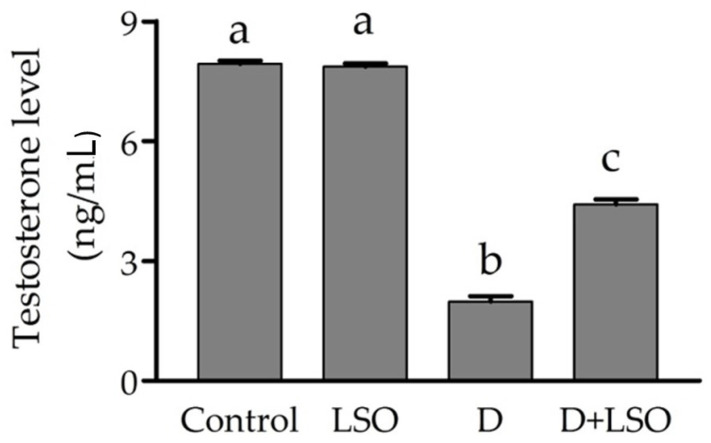
Effect of LSO on serum testosterone levels in diabetic mice with STZ-induced testicular toxicity. Data are expressed as the mean ± SE (n = 6 per group). Bars with different letters are significantly different from each other (*p* < 0.05).

As shown in Figure 2, STZ-induced diabetes led to a significant decrease (−75%) in serum testosterone (T) in the diabetic mice compared to the control group. Meanwhile, the administration of LSO to the diabetic mice produced a significant increase (+128.5%) in serum testosterone levels compared to the diabetic mice group. However, serum testosterone was not completely restored to normal levels, and a significant decrease was observed in the D+LSO mice compared to the control and LSO groups.

### 2.3. Redox Status in the Testes of Diabetic Mice Treated with/without LSO

**Figure 3:**
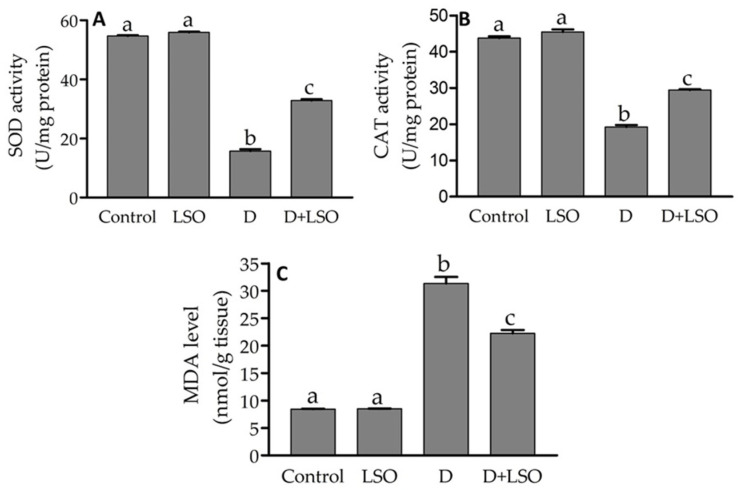
Effect of LSO on the testicular biomarkers of oxidative stress: (**A**) SOD; (**B**) CAT; and (**C**) MDA in diabetic mice with STZ-induced testicular toxicity. Data expressed as the mean ± SE (n = 6 per group). Bars with different letters are significantly different from each other (*p* < 0.05).

The activities of the testicular enzymatic antioxidants SOD and CAT in the diabetic group demonstrated significant decreases of −71.2% and −55.9%, respectively, compared to the control group (Figure 3A,B). Conversely, treatment of the diabetic mice with LSO resulted in a significant increase in SOD (+108.4%) and CAT (+52.6%) activities compared to the diabetic mice. Moreover, it was observed that the testicular MDA level was significantly increased (+270.7%) in the diabetic mice compared to that of the control. On the other hand, the MDA level in the D+LSO group was significantly decreased (−28.9%) compared to the diabetic mice (Figure 3C).

### 2.4. NF-kB and Pro-Inflammatory Cytokines in the Testes of Diabetic Mice Treated with/without LSO

**Figure 4:**
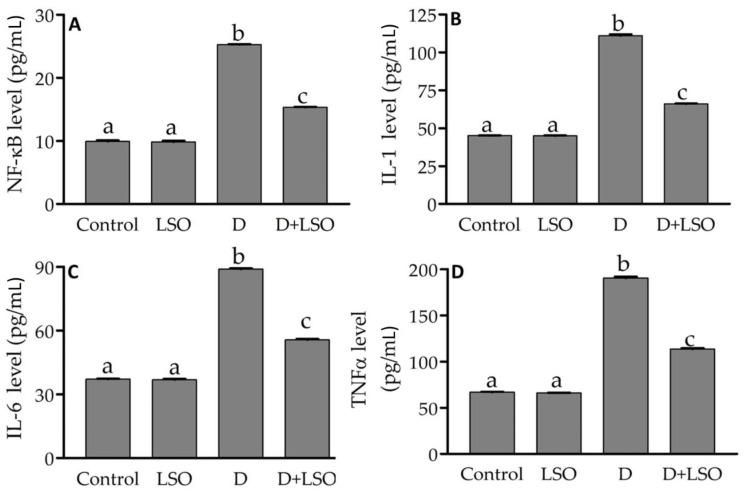
Effect of LSO on the testicular protein levels of NF-kB and the pro-inflammatory cytokines in the diabetic mice with STZ-induced testicular toxicity. (**A**) NF-kB; (**B**) IL-1; (**C**) IL-6; and (**D**) TNF-α. The results are expressed as the mean ± SE (n = 6 per group). Bars with different letters are significantly different from each other (*p* < 0.05).

To assess the anti-inflammatory role of LSO, the testicular protein level of the NF-kB p65 subunit was determined using ELISA, as shown in Figure 4A. It was significantly increased (+254.7%) in the diabetic mice compared to the control and LSO. However, it was significantly decreased (−39.4%) in the D+LSO-treated group compared to the diabetic one. In addition, the level of testicular pro-inflammatory cytokines, IL-1, IL-6, and TNF-α, was also assessed. Our data showed a significant increase in the levels of IL-1 (+146.5%), IL-6 (+139.1%), and TNF-α (+184.4%) in the diabetic mice compared to the control (Figure 4B–D). Conversely, in the D+LSO mice, treatment of the diabetic mice with LSO significantly reduced IL-1 by −40.5%, IL-6 by −37.4%, and TNF-α by −40.3% when compared to the corresponding levels in the diabetic group.

### 2.5. NF-kB p65 Subunit Expression in the Testes of Diabetic Mice Treated with/without LSO

**Figure 5:**
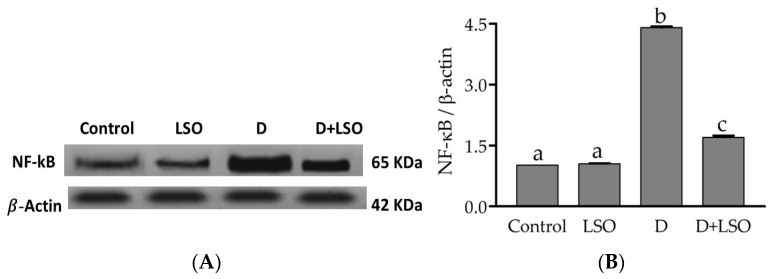
(**A**) Effect of LSO on testicular NF-kB expression in diabetic mice with STZ-induced testicular toxicity assessed by Western blot analysis. (**B**) The quantification histogram of NF-kB p65 protein expression normalized by β-actin. The data are expressed as the mean ± SE (n = 6 per group). Bars with different letters are significantly different from each other (*p <* 0.05).

To further confirm the involvement of NF-kB in the pathway via which LSO modulates the inflammatory response, the expression of testicular NF-kB was assessed using Western blot analysis (Figure 5A). NF-kB p65 subunit expression was notably upregulated in the diabetic mice compared to the control and LSO groups (by +76.81% and +76.13%, respectively). Whereas downregulation of NF-kB expression was observed upon LSO administration in the D+LSO mice by −31.12% compared to the diabetic mice (Figure 5B). The data suggested the impact of the NF-kB pathway on the LSO therapeutic action against testicular damage in the diabetic mice model.

### 2.6. Effect of LSO on Testicular Histoarchitecture in the Diabetic Mice Treated with/without LSO

**Figure 6:**
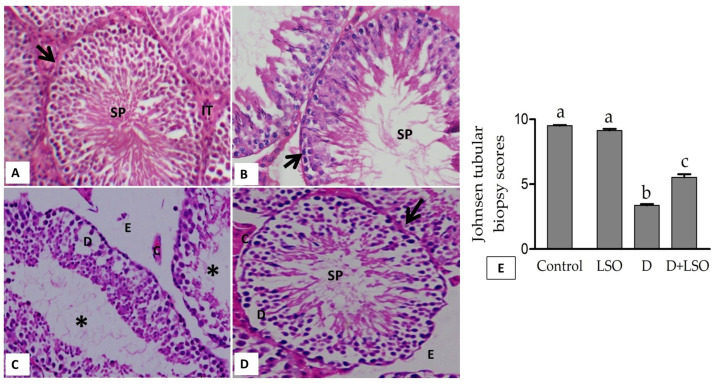
Light micrographs of testicular tissues of the experimental groups. Control group (**A**) exhibiting a regular seminiferous tubule with complete spermatogenic series, the LSO group (**B**) showing a normal spermatogenic pattern, the diabetic group (**C**) demonstrating marked tubular atrophy and interstitial edema, the D+LSO group (**D**) indicating noticeable preservation in seminiferous tubule histoarchitecture, and (**E**) the JTBS score. Bars with different letters are significantly different (*p* < 0.05) between groups. Hematoxylin and eosin (H, E) staining, original magnification 400×. Spermatozoa (SP), seminiferous tubules (arrows), interstitial tissue (IT), atrophic seminiferous tubules (asterisks), desquamation (D), edema (E), and congestion (C).

Figure 6 shows the representative photomicrographs and histopathological scoring of testes in all studied groups. Seminiferous tubules with normal spermatogenesis stages were observed in the control group (Figure 6A). The LSO group revealed no changes similar to the control (Figure 6B). STZ-induced diabetes resulted in marked damage in the seminiferous epithelium, and empty/atrophied tubules were predominant in this group. Germinal cell death was also notable. In addition, there was obvious edema in the interstitial space and sloughing (detachment) in the germinal epithelial layer (Figure 6C). Conversely, the testes of the D+LSO mice exhibited little degeneration in the interstitial space and lesser detachment in the germinal layer, and the tubular lumen was filled with spermatids, indicating improvement in spermatogenesis series. However, edema, congestion, and few epithelial detachments were still found (Figure 6D). The mean JTBS (Figure 6E) in the diabetic group (D) significantly decreased by −64.5% compared to the control group, revealing atrophy and hypospermatogenesis. However, treatment of the diabetic mice with LSO significantly increased the mean JTBS value by +63.85% in the D+LSO group compared to that recorded in the diabetic (D) group alone.

## 3. Discussion

Diabetes mellitus (DM) is a recognized cause of male sexual dysfunction, which may lead ultimately to sub/infertility. Nearly half of diabetic men have reduced semen quality and exhibit reproductive dysfunction [9]. The underlying mechanisms involve, among others, chronic hyperglycemia-induced oxidative stress, inflammation, neuropathy, and insulin resistance (IR), resulting in a reduction in the level of reproductive hormones (FSH, LH, and testosterone) as well as structurally injured male reproductive organs [9,79]. Thus far, the treatment of DM-associated male reproductive dysfunction is challenging, as no established medication that specifically addresses the reproductive health of diabetic patients is available [9]. Knowing that OS is a main cause of damaged reproductive organs, natural products (NPs) with antioxidant capacity can thus be used as supplements for the prevention and/or treatment of diabetic complications, including reproductive dysfunction. To our knowledge, the current study is the first to demonstrate the protective effect of *L. sativum* seed oil (LSO) against diabetic-induced male reproductive dysfunction. LSO exhibited antioxidant activity as it restored normal levels of enzymatic antioxidants and oxidative stress biomarkers. Moreover, LSO showed anti-inflammatory effects via modulating the levels of inflammatory molecules, including NF-kB, IL-1, IL-6, and TNF-α. In addition, LSO was able to restore normal levels of testosterone.

GC-MC spectral analysis revealed the presence of ten bioactive components in LSO, the major of which were two forms of vitamin E, namely γ-tocopherol (62.1%) and δ- tocopherol (3.91%), followed by six alkanes (24.22%), the antioxidant phenol derivative butylated hydroxytoluene (BHT; 8.04%), and an alcoholic compound (1.73%). Many of these bioactive components possess antioxidant and/or anti-inflammatory activities and hence a wide range of applications, including potential therapeutic benefits. Our data are consistent with those of Umesha and Naidu, who found that feeding rats on a diet with LSO increased blood tocopherol levels and elevated the activity of antioxidant enzymes [80]. Indeed, forms of vitamin E such as γ-tocopherol (γT), δ-tocopherol (δT), and γ-tocotrienol (γ-T3) are strong natural therapeutic antioxidants and known to possess anti-inflammatory effects against various diseases with an underlying inflammatory, redox, or malignant component [81,82]. Furthermore, butylated hydroxytoluene (BHT) is a lipophilic organic substance widely used to prevent free radical-mediated oxidation in organic fluids, and its therapeutic implications have been previously suggested [83,84,85]. In addition to the above-mentioned components, the 2-methyltetracosane alkane detected in the LSO extract is known to possess free radical scavenging activity and proposed antibacterial activity [86,87]. Also, LSO contains tetracosane which has previously been reported as an antioxidant and anticancer alkane compound [88]. The presence of such bioactive compounds in LSO may be a key factor in reducing the free radicals resulting from hyperglycemia in STZ-induced diabetic mice. Oxidative stress is a key mechanism associated with the onset of diabetic issues. In diabetes, hyperglycemia increases mitochondrial glucose oxidation which results in ROS release into the cell cytoplasm, promoting pro-oxidants over antioxidants [35,89,90]. In the present research, the administration of LSO to the STZ-induced diabetic mice impeded OS in testicular tissues, as evidenced by the increase in the enzymatic activities of CAT and SOD and the decrease in the levels of MDA. Similarly, other reports have shown the potent antioxidant properties and improvement in testicular antioxidants exerted by other seed oils via increasing the overall levels of endogenous antioxidants [91,92].

Indeed, inflammation is known to play an emerging role in the pathophysiology of diabetes, whereby various inflammatory pathways and biomarkers of inflammation are involved [93]. Moreover, it has been previously reported that the innate immune and inflammatory responses are involved in the progression of hyperglycemia-induced secondary diabetic complications [94]. The transcription factor nuclear factor kappa B (NF-kB) plays a major role in innate immunity and inflammatory responses. Moreover, it is also involved in the modulation of responses to various stimuli, including free radicals and cytokines [95,96]. Remarkably, chronic hyperglycemia and the concurrent increased levels of advanced glycation end products (AGEs) and their receptors (RAGE) as well as OS activate NF-kB by various pathways that subsequently trigger the expression of various pro-inflammatory cytokines, including IL-1, IL-6, and TNF-α, which are crucial for the development of inflammation in diabetes and other chronic diseases [97,98,99]. Our data revealed a substantial increase in the levels of testicular inflammatory markers, NF-kB, IL-1, IL-6, and TNF-α, in the diabetic mice compared to the control. In accordance with our findings, previous studies have reported high levels of inflammation in diabetic rat testes [100,101,102]. Notably, the administration of LSO to the diabetic mice resulted in anti-inflammatory effects, as indicated by reduction in the elevated pro-inflammatory cytokines in the D+LSO mice, a modulatory effect associated with decline in NF-kB expression.

Previous studies have reported the anti-inflammatory effects of LSO [73,103,104], which is probably attributed to the inhibition of the NF-kB signaling pathway. Moreover, vitamin E forms, representing the major components in LSO extracted in the current study, scavenge reactive nitrogen species (RNS) and suppress pro-inflammatory signaling such as NF-kB [105,106]. In addition, it has been reported that butylated hydroxytoluene (BHT) and γ-tocopherol (γT) contribute to *L. sativum*’s anti-inflammatory, antioxidant, and immunomodulatory properties [65].

It has been demonstrated that spermatogenesis and steroidogenesis are inhibited by hyperglycemia-induced OS and increased levels of pro-inflammatory markers, IL-1 and TNF-α [56,107,108]. Moreover, previous in vitro and in vivo studies have revealed the inhibition of testosterone (T) production induced by LH consequent to the inhibitory effect exerted by IL-1 and TNF-α on Leydig cells [109,110]. In line with these findings, a significant decrease in serum testosterone levels in the STZ-induced diabetic mice was observed compared to the control. Treatment with LSO markedly restored testosterone levels in the D+LSO mice compared to the diabetic group. Comparably, improvement in testosterone levels in diabetic animal models has been previously reported upon treatment with seed oil extracted from various medicinal plants [91,92]. The restoration of the serum testosterone levels in the diabetic mice treated with LSO (D+LSO) when compared to the STZ-induced diabetic mice affirms the therapeutic potential of LSO on testicular tissues in diabetic mice. Owing to the reported antioxidant and anti-inflammatory effects of LSO [73,80,111], the protective effect of LSO on testicular tissues is attained via the reduction of oxidative damage and suppression of inflammation induced by hyperglycemia. Moreover, STZ induction of diabetes resulted in damaged testicular tissue and hypospermatogenesis as indicated by JTBS. This may be explained by the hyperglycemia-induced increased generation of free radicals and the reduced activity of antioxidant enzymes in the testicular tissues, the elevated levels of pro-inflammatory markers, the suppression of gonadotropin and testosterone release, and the inhibition of steroidogenesis [91,112,113].

## 4. Materials and Methods

### 4.1. Plant Material

The *Lepidium sativum* L. seeds were purchased from a local market in Hofuf city, Al-Ahsa, Saudi Arabia.

### 4.2. Extraction of Fixed Oil

The *Lepidium sativum* L. seeds were ground using an electrical grinder to a fine powder; then, 470 g of the seed powder was extracted with petroleum ether (40–60 °C):diethyl ether (1:1 *v*/*v*) until it was clear using the soxhlet apparatus at 40 °C. The solvent was removed under reduced pressure. The total fixed oil was quantified as previously described [114] and kept at 4 °C for further analysis.

### 4.3. Gas Chromatography-Mass Spectrometry (GC-MS) Analysis of LSO

The chemical composition of LSO was identified using a GCMS-QP2010 SE gas chromatograph-mass spectrometer (SHIMADZU, Kyoto, Japan) with a direct capillary column Rtx–5MS (30 m × 0.25 mm × 0.25 µm film thickness). The temperature of the column oven was first maintained at 50 °C (2 min hold), then raised by 10 °C/min to 150 °C, and after that to 300 °C by 10 °C/min. The injector temperature was maintained at 250 °C. At a constant flow rate (1 mL/min), helium was employed as a carrier gas. Samples of 1.0 µL were inoculated automatically by an AOC-20S Auto sampler attached with GC in the splitless mode. Electron ionization (EI) mass spectra were obtained at 70 eV ionization voltages over the range of *m*/*z* 20–500 in full scan mode. The temperatures of the ion source and transfer line were set to 250 and 300 °C, respectively. The constituents were recognized by comparing their retention times and mass spectra with those of the NIST 05 mass spectral database.

### 4.4. Animals

Twenty-four adult (8 weeks old) male mice (25~28 g) obtained from an animal facility at King Faisal University, Saudi Arabia, were used in this study. The mice were main-tained under standard conditions with a 12 h light/dark cycle, a temperature of (~20–22 °C), and 40–60% relative humidity. The animals were given regular food with water ad libitum. All experimental procedures were followed according to relevant guidelines and regulations for experimental animal use in research established by the Deanship of Scientific Research, Vice Presidency for Graduate Studies and Scientific Research, King Faisal University (KFU-REC-2023-MAR-ETHICS734).

### 4.5. Induction of Diabetes

Overnight-fasted mice were induced by a single intraperitoneal (i.p.) injection of 50 mg/kg in 0.1 mol/L citric acid buffer, pH 4.5 streptozotocin (STZ; Sigma, St. Louis, MO, USA). The diabetic state was confirmed within 7 days after STZ injection by determining tail vein blood glucose levels. Mice with a non-fasting blood glucose concentration over 16.67 mmol/L for three consecutive days were diagnosed as diabetic models [115].

### 4.6. Experimental Design

After two weeks of diabetes induction, the experimental mice were separated into four groups (6 mice in each).

- Control: The mice were injected intraperitoneally (i.p.) with citric acid–sodium citrate buffer solution.
- Diabetic (D): Diabetes was induced by a single intraperitoneal (i.p.) injection of streptozotocin (STZ; 50 mg/kg/day) for five consecutive days [115].
- Lepidium seed oil (LSO): The mice were administrated intragastrically with 0.5 mL/kg LSO twice a week for four weeks.
- D+LSO: The diabetic mice administrated with LSO as mentioned in the LSO-alone group.

### 4.7. Tissue Sampling

The mice were euthanized under anesthesia and sacrificed to their collect blood. The blood samples were collected and centrifuged at 5000× *g* for 10 min; the separated sera were subjected to testosterone analysis. Immediately, the paired testes were excised. The right testes were stored in 10% formaldehyde fixative fluid for histomorphology, whereas the left testes were stored at −80 °C for biochemical and molecular (western blotting) analyses.

### 4.8. Assay of Serum Testosterone

Serum samples were used to determine the levels of testosterone (T) using a mouse testosterone enzyme-linked immunosorbent assay (ELISA) kit (Cat. No. MBS494055; MyBioSource, Inc., San Diego, CA, USA) according to manufacturer’s instructions. Spectrophotometric analysis was used to evaluate the optical density at 450 nm, and the testosterone level (ng/mL) was evaluated against the reference stranded assessed in a similar manner.

### 4.9. Assessment of Redox Status

The colorimetric assay kits for determining the antioxidant enzymes catalase (CAT; Cat. No. CA 2516) and superoxide dismutase (SOD; Cat. No. SD 2520) as well as the LPO biomarker, malondialdehyde (MDA; Cat. No. MD 2528), were purchased from Bio Diagnostic (Dokki, Giza, Egypt). The tissues from the left testes were homogenized in 0.1 M phosphate buffer (pH 7.4). The homogenates were centrifuged at 2800× *g* for 30 min at 4 °C. The obtained supernatants were utilized for biochemical estimations of CAT, SOD, and MDA as per the guidelines provided by the manufacturer. The protein content in the testicular homogenates was determined as previously described [116], employing bovine serum albumin (BSA) as the standard.

### 4.10. Determination of NF-kB Factor and Pro-Inflammatory Cytokines

The testicular protein levels of nuclear factor-kappa B (NF-kB) p65 subunit, interleukin-1 (IL-1), interleukin-6 (IL-6), and tumor necrosis factor-alpha (TNF-α) were measured using ELISA kits (NF-kB, Cat. No.: orb409109; Biorbyt Ltd., Cambridge, UK) and (IL-1, Cat. No.: MBS264984; IL-6, Cat. No., MBS843429; TNF-α, Cat. No.: MBS825002; My Bio-Source, Inc., San Diego, CA, USA), respectively, in accordance with the manufacturer’s instructions.

### 4.11. Western Blotting

Approximately 50 mg of testicular tissues were added to RIPA lysis buffer (Cat. No.: PL005, BIO BASIC INC., Markham, ON, Canada). The tissue samples were homogenized, and protein quantifications were carried out by a Bradford protein assay kit (Cat. No.: SK304, BIO BASIC INC., Markham, ON, Canada). Equal quantities of protein per specimen were exposed to 10% sodium dodecyl sulfate–polyacrylamide gel electrophoresis (SDS-PAGE), and the resolved proteins were electrophoretically transmitted onto polyvinylidene difluoride (PVDF) membranes using the Trans-Blot Turbo Transfer System (Bio-Rad Laboratories, Inc., CA, USA). These membranes were blocked in tris-buffered saline with 0.1% Tween 20 (TBST) and 3% BSA for 60 min at room temperature. Subsequently, the membranes were probed overnight at 4 °C with primary antibodies against NF-kB p65 (Cat. No. ab31481, 1:1000) and β-actin as an internal loading control (Cat. No. ab8226, 1:10,000), both purchased from Abcam (Cambridge, UK). After washing with TBST, the membranes were incubated with horseradish peroxidase-conjugated goat anti-rabbit IgG secondary antibodies at room temperature for 1 h. The blots were washed three times between the primary as well as secondary antibody incubations. Finally, the immunoreactive bands of the NF-kB p65 subunit were imaged using the ChemiDoc MP imager with Image Lab^TM^ software (version 6.1) (Bio-Rad Laboratories, Inc., Hercules, CA, USA) to validate the Western blot data via total protein normalization by the β-actin housekeeping protein.

### 4.12. Histological Study on the Testes

The excised right testes were fixed in 10% formaldehyde solution at 28 °C for 24 h, dehydrated, and inserted in paraffin wax. Traditional H&E (hematoxylin and eosin) dye was used to stain the thin sections (4 μm thick). A typical light microscope (Nikon Corporation, Tokyo, Japan) was used for examining the slides. Twenty seminiferous tubules from each sample were magnified at 400× to determine the testicular maturity and damage using the Johnsen’s tubular biopsy score (JTBS) method [117]. Each seminiferous tubule was given a score between 1 and 10 to indicate its level of germinal epithelial maturity according to the following description: score (1), no cells appear in the tubular section; score (2), no germ cells exist; score (3), just the spermatogonia can be seen; score (4), limited primary spermatocytes; score (5), numerous primary spermatocytes but no spermatozoa or spermatids; score (6), only a few spermatids; score (7), a large number of early spermatids deprived of differentiation; score (8), a few differentiated spermatids; score (9), several differentiated spermatids; and finally, score (10), regular tubules with lots of sperms.

### 4.13. Statistical Analyses

The results were expressed as the mean ± standard error (SE). The sample size (n = 6) was identified using the G power program (version 3.1.9.7). Graph Pad prism software(version 4.03, San Diego, CA, USA) was used for the statistical analysis. Using the Shapiro–Wilk normality test, the data’s normality was evaluated. One-way ANOVA was used to analyze the statistical variance between the groups, and Tukey’s post hoc test was used to compare the groups. Statistics were determined to be significant for all *p* values < 0.05.

## 5. Conclusions

**Figure 7:**
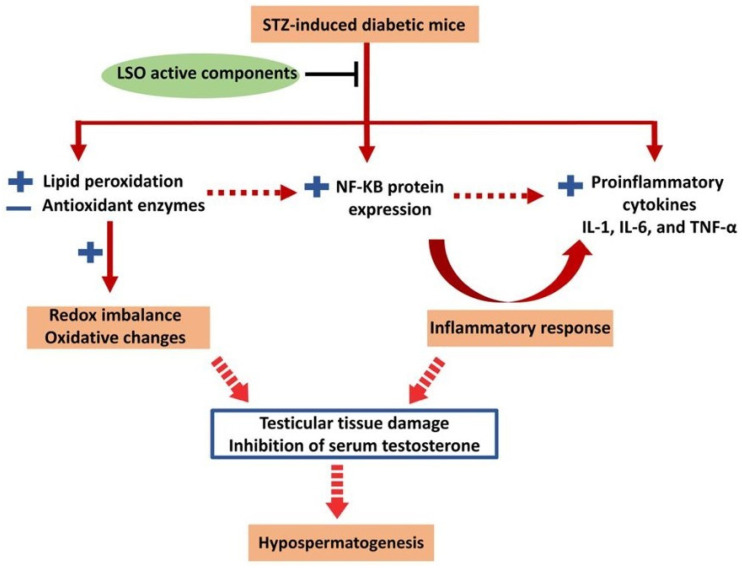
Proposed mechanisms underlying the therapeutic effects of LSO against testicular damage and dysfunction in diabetic mice.

The obtained results show, for the first time, the protective effect of LSO against male reproductive dysfunction observed in diabetic mice. Alleviation of diabetic-induced reproductive dysfunction might be attributed to the improvement in the testicular antioxidant defense system, lowering the levels of pro-inflammatory markers, and the restoration of serum testosterone levels (Figure 7). The findings of the current study support the previously reported antioxidant and anti-inflammatory activities of LSO and pave the way for its therapeutic application in the management of diabetes-associated male reproductive dysfunction. We believe that administering LSO to male diabetic patients will effectively ameliorate the process of spermatogenesis and enhance the architecture of the testicular tissue.

## Figures and Tables

**Table 1:** Chemical components identified in LSO using GC-MS spectral analysis.

Peak No.	Retention Time (RT)	Area%	Compound Name
1	14.72	8.04	Butylated Hydroxytoluene
2	17.032	1.82	Tridecane, 1-iodo-
3	18.153	2.72	2-methyloctacosane
4	19.142	3.91	*δ*-Tocopherol
5	20.123	4.81	10-Methylnonadecane
6	25.975	2.67	2-methyltetracosane
7	26.325	1.73	Z-6-Pentadecen-1-ol acetate
8	27.393	8.12	2-methylhexacosane
9	28.701	62.1	γ-Tocopherol
10	29.041	4.08	Tetracosane, 1-bromo-

## Data Availability

All data analyzed during this study are included in this published article.
